# Meningioma surgery in younger and older adults: patient profile and surgical outcomes

**DOI:** 10.1007/s41999-017-0015-1

**Published:** 2017-12-21

**Authors:** K. Mariam Slot, Jocelyne V. M. Peters, W. Peter Vandertop, Dagmar Verbaan, Saskia M. Peerdeman

**Affiliations:** 1 Neurosurgical Center Amsterdam, VU University Medical Center, PO Box 7057, 1007 MB Amsterdam, The Netherlands; 2 Neurosurgical Center Amsterdam, Academic Medical Center, Amsterdam, The Netherlands

**Keywords:** Meningioma, Elderly, Surgery, Preoperative factors, Outcome, Adults

## Abstract

**Background:**

Due to increasing life expectancy, the number of older patients harboring a meningioma is expected to increase. We determined whether preoperative variables and postoperative clinical outcome differ between younger and older adults.

**Methods:**

Medical records of meningioma patients were retrospectively analyzed. Preoperative variables were age, gender, neurological symptoms, Karnofsky Performance Status (KPS), American Society of Anesthesiologists Physical Status (ASA)-classification and tumor characteristics. Clinical outcome was assessed using complication rates, length of hospital stay and destination after discharge. After 6–12 and 12–18-month KPS, neurological symptoms and Glasgow Outcome Scale (GOS) scores were assessed for older (age ≥ 65 years) and younger adults (18–65 years) using Mann–Whitney *U*, *T* test, Pearson’s Chi square or Fisher’s exact.

**Results:**

89 patients were included (23 ≥ 65 years). Before surgery, older patients scored higher on ASA classification (*p* = 0.003) and lower on KPS (*p* = 0.017). There was no significant difference postoperatively in mortality, complications and duration of hospital stay. Less older patients were discharged directly to home compared to younger adults (52 vs 80%, respectively; *p* = 0.004). In surviving patients, less older subjects had a good recovery (GOS 4–5) at 6–12 months’ follow-up compared to younger subjects (64 vs 93%, respectively; *p* = 0.035). At 12–18 months, there was no significant difference in good recovery between both age groups (82 vs 92%).

**Conclusion:**

In this cohort, outcome was worse for patients ≥ 65 years old in terms of discharge destination and good recovery at 6–12 months. At 12–18 months follow-up, older subjects performed not significantly different from younger ones. Careful patient selection seems essential to reach good results in meningioma surgery for patients ≥ 65 years old.

## Introduction

The incidence rate of meningiomas increases with age, ranging from 26 to 51 per 100,000 patients [1]. Due to wider use of imaging techniques and an increasing life expectancy, meningiomas are expected to be diagnosed more frequently [2, 3]. This raises the question whether surgical excision, as first therapeutical option, is as valid for older patients as it is for younger adult patients, and whether the postoperative clinical outcome in these patients, in terms of complications, morbidity and mortality, is comparable to that in younger patients.

As in general surgery, studies regarding the outcome of older patients undergoing meningioma surgery have shown conflicting data, which may be attributed to comorbid conditions [4–20]. The aim of this study is to determine whether preoperative variables and postoperative clinical outcome differ between age groups after meningioma surgery.

## Materials and methods

### Patient population

Patients who underwent meningioma surgery at the VU University Medical Center (Amsterdam, the Netherlands), between January 2005 and June 2012 were selected from the hospital database. Patients with a pathologically confirmed World Health Organization (WHO) grade I meningioma were included. Symptomatology was the main indication for surgery, followed by radiological aspects of the tumor. Patients with multiple meningiomas were included if surgery was the only therapy performed. Exclusion criteria were age under 18 years, neurofibromatosis, other intracranial tumors, or radiotherapy directly following surgery. In total, 89 patients were included.

### Data collection

The following data were retrospectively obtained from the medical records: age, gender, neurological symptoms, preoperative Karnofsky Performance Status (KPS), American Society of Anesthesiologists Physical Status Classification (ASA score), tumor location and presence of peritumoral edema on preoperative MR imaging [21, 22]. Postoperative outcome was assessed using the following data: (1) length of hospital stay, (2) destination after discharge, (3) complications within 30 days, and (4) mortality within 30 days. Complications were defined as “unintended and unwanted events or conditions during, or within, 30 days following medical treatment, so harmful for the patient’s health that adjustment of medical treatment is needed, or that irreversible damage occurs” [23]. Complication data were collected from the prospective complication database of the department of neurosurgery. Included were neurosurgical complications (i.e., postoperative hemorrhage, wound dehiscence, infection, seizures, CSF leakage, hygroma, hydrocephalus or neurological deficits), as well as systemic complications (i.e., pressure ulcers, multiple organ failure, infections, cardiac arrhythmia, heart failure, thrombo-embolic events, respiratory insufficiency, gastrointestinal complications, kidney failure or metabolic disorders). Urinary tract infection, pneumonia, sepsis and meningitis were defined as clinical symptoms followed by a positive culture. Severity of complications was graded according to the Classification of Surgical Complications according to Dindo [24].

The long-term outcome was assessed at 6–12 months and at 12–18 months after surgery using (1) Glasgow Outcome Scale score (GOS), (2) KPS score and (3) neurological functioning by determining whether the symptoms had resolved, stabilized, improved or worsened at follow-up [25].

### Statistical analysis

Patients were divided into older (age ≥ 65 years) and younger adult (age 18–65 years) patients. Since older patients are becoming more frequent in clinical practice an additional subgroup analysis of patients over 70 years old was performed. Furthermore, a subgroup analysis for different ASA groups was done. Continuous variables were tested on normal distribution. Differences between age groups in pre- and postoperative factors were analyzed with Mann–Whitney *U* (not normally distributed continuous variable), *T* test (normally distributed continuous variable), Pearson’s Chi-square (dichotomous or categorical variable with a minimum expected count > 5) or Fisher’s exact test (dichotomous or categorical variable with a minimum expected count < 5) using SPSS Statistics 22.

The percentages of follow-up data were calculated from the total number of available follow-ups of both groups. An additional analysis comparing long-term GOS scores (6–12 months, as well as 12–18 months) between age groups was performed in only those patients who survived the period to the specific time of follow-up.

## Results

### Preoperative factors

Symptomatology was the main indication for surgery, followed by radiological aspects of the tumor. Clinical characteristics of the 89 included patients are summarized in Table 1. There was no significant difference in indications for surgery or location of the tumor between the age groups (Table 1). Older patients had higher ASA scores (*p* = 0.003) and lower KPS scores (*p* = 0.017) compared to the younger adults. None of the patients in this cohort had a preoperative KPS lower than 50.

**Table 1:** Clinical characteristics

	*N* (%)	18–65 years	≥65 years	*p*
Patients	89 (100)	66 (74)	23 (26)	
Gender				
Female	61 (69)	45 (68)	16 (70)	NS
Age (years)^†^				
Mean (SD)	54.4 (12)	49.3 (9)	69.2 (5)	0.008
Preoperative KPS				
≤ 60	10 (12)	4 (6)	6 (26)	0.017
≥ 70	79 (89)	62 (94)	17 (74)	
ASA				
I	21 (24)	19 (29)	2 (9)	0.003
II	55 (62)	42 (64)	13 (57)	
III	13 (15)	5 (8)	8 (35)	
Tumor location^‡^				
Convexity	33 (37)	24 (36)	9 (39)	NS
Falx/tentorium	16 (18)	11 (17)	5 (22)	
Skull base	14 (16)	13 (20)	1 (4)	
Posterior fossa	7 (8)	6 (9)	1 (4)	
Other locations	6 (7)	4 (6)	2 (9)	
Multiple tumors	13 (15)	8 (12)	5 (22)	
Preoperative PTBE				
Yes	41 (46)	26 (39)	15 (65)	NS
Indication for surgery				
Progressive deficit	26 (29)	18 (27)	8 (35)	NS
Epilepsy	15 (17)	10 (15)	5 (22)	
Tumor growth	16 (18)	13 (20)	3 (13)	
Size of tumor	9 (10)	7 (11)	2 (9)	
New symptoms	6 (7)	3 (5)	3 (13)	
PTBE	6 (7)	5 (8)	1 (4)	
Other	5 (6)	5 (8)	0 (0)	
Focal neur. deficit	5 (6)	4 (6)	1 (4)	
Unknown	1 (1)	1 (2)	0 (0)	

*KPS* Karnofsky Performance Status, *ASA* American Society of Anesthesiologists’ Physical Status Classification, *PTBE* peritumoral brain edema (PTBE was unknown in *N* = 1 of the adult group), *NS* not significant

^†^Statistical analysis: *t* test was used

^‡^Some patients had one or more meningiomas, which covered more than one location

### Postoperative outcome

Data on postoperative outcome within 30 days after surgery are presented in Table 2. Mortality, complications, and duration of hospital stay did not differ between younger and older subjects. The mortality rate was 2%: two female patients younger than 65 years of age died within 30 days postoperatively. One patient due to postoperative parenchymal hemorrhage in the midbrain after partial resection of a sphenopetroclival meningioma. The other patient had postoperative cerebrospinal fluid leakage, followed by meningitis, seizures, encephalopathy and respiratory insufficiency. One male patient (69 years old) died in the follow-up period between 12 and 18 months (528 days after surgery). The cause of death was unknown.

**Table 2:** Postoperative outcome

	*N* (%)	18–65 years	≥65 years	*p*
Patients	89 (100)	66 (74)	23 (26)	
Complications*				
None	42 (47)	33 (50)	9 (39)	NS
Grade I	21 (24)	13 (20)	8 (35)	
Grade II	8 (9)	6 (9)	2 (9)	
Grade IIIa	4 (5)	3 (5)	1 (4)	
Grade IIIb	8 (9)	6 (9)	2 (9)	
Grade IV	4 (5)	3 (5)	1 (4)	
Grade V	2 (2)	2 (3)	0 (0)	
Perioperative mortality	2 (2)	2 (3)	0 (0)	NS
Discharge location^†^				
Home	65 (73)	53 (80)	12 (52)	0.002
Rehabilitation	2 (2)	2 (3)	0 (0)	
Other hospital	17 (20)	9 (14)	8 (35)	
Nursing home	3 (3)	0 (0)	3 (13)	
Duration of hospital stay^‡^				
Median (IQ range)	4 (3–6)	4 (3–6)	4 (4–7)	NS

*NS* not significant

*In case of multiple complications per patient: the highest grade of complications is noted

^†^The group of discharged adult patients consists of 64 patients because 2 patients deceased during their admission

^‡^Amount of days in the academic hospital. Statistical analysis: Mann–Whitney *U* test

The median (interquartile range) length of stay in the hospital was 4 (3–6) days. Of the younger adults, 80% were discharged directly to home, compared to only 52% of the older patients (*p* = 0.004). In the 65 + group, eight (35%) patients were transferred to another hospital, whereas for younger adults this was 14%. Five of those eight patients were transferred to another hospital because they needed minor care and home care was not organized yet. Two had a psychiatric history and needed longer observation of mental care, and one had neurological deficits and was evaluated for a nursing home. None of the 65 + patients were transferred to a medical rehabilitation center.

Subgroup analysis for patients in ASA group I, II and III separately showed no significant difference in complication rate and perioperative mortality between younger and older adults. Discharge location was significantly different between both age groups for ASA II patients (*p* = 0.006). The median (IQ range) duration of hospital stay was 4 (3–5) days and 5 (4–5) days for 18–65 and ≥ 65 years old ASA I patients, respectively (*p* = 0.857). For ASA II, this was 4 (3–6) days and 5 (4–10) days (*p* = 0.036), and for ASA III 6 (3–9) days and 4 (3–6) days for 18–65 and ≥ 65 years old, respectively (*p* = 0.683).

No significant difference in complication rate and perioperative mortality between patients 18–70 and ≥ 70 years old was found. The discharge location was significantly different between those age groups (*p* = 0.003), which is comparable to the findings for patients under and over 65 years old. The median (IQ range) duration of hospital stay was 4 (3–6) days and 7 (5–18) days for 18–70 and ≥ 70 years old, respectively.

### Long-term outcome

Long-term follow-up data were available in up to 60% of the patients. They are presented in Table 3. Neurological symptoms, KPS and GOS scores did not differ between the two age groups at both times of follow-up. In the subgroup analysis for different ASA groups, we found a significant difference between both age groups for KPS at 12–18 months (*p* = 0.032) for ASA II patients.

**Table 3:** Long-term outcome

	*N* (%)	18–65 years	≥65 years	*p*
Total number of patients	89 (100)	66 (74)	23 (26)	
Symptoms 6–12				
New/worse	6 (7)	5 (8)	1 (4)	NS
Persistent	10 (11)	7 (11)	3 (13)	
Improved	16 (18)	10 (15)	6 (26)	
None	10 (11)	9 (14)	1 (4)	
Missing data	47 (53)	35 (53)	12 (52)	
Symptoms 12–18				
New/worse	11 (12)	9 (14)	2 (9)	NS
Persistent	12 (13)	9 (14)	3 (13)	
Improved	8 (9)	6 (9)	2 (9)	
None	19 (21)	15 (23)	4 (17)	
Missing data	39 (44)	27 (41)	12 (52)	
KPS 6–12 months				
≤ 60	2 (2)	0 (0)	2 (9)	NS
≥ 70	39 (44)	30 (45)	9 (39)	
Missing data	48 (54)	36 (55)	12 (52)	
KPS 12–18 months				
≤ 60	3 (3)	1 (2)	2 (9)	NS
≥ 70	47 (53)	38 (58)	9 (39)	
Missing data	39 (44)	27 (41)	12 (52)	
GOS 6–12 months				
Death	2 (2)	2 (3)	0 (0)	NS
Moderate disability	6 (7)	2 (3)	4 (17)	
Good recovery	35 (39)	28 (42)	7 (30)	
Missing data	46 (52)	34 (52)	12 (52)	
GOS 12–18 months				
Death	3 (3)	2 (3)	1 (4)	NS
Moderate disability	5 (6)	3 (5)	2 (9)	
Good recovery	45 (51)	36 (55)	9 (39)	
Missing data	36 (40)	25 (38)	11 (48)	

*p* values were calculated from the total number of available follow-ups

*NS* not significant, *KPS* Karnofsky Performance Status, *GOS* Glasgow Outcome Score

An additional analysis in surviving patients, as presented in Table 4, showed a difference in good and moderate recovery at 6–12 months postoperatively between younger and older patients (93 and 7 vs 64 and 36%, respectively, *p* = 0.035). This difference in good and moderate outcome of surviving patients was not present at follow-up after 12–18 months (92 and 8% in younger adults vs 82 and 18% in the older subjects, respectively; *p* = 0.301).

**Table 4:** Long-term GOS for surviving patients only

	*N* (%)	18–65 years	≥65 years	*p*
Number of patients at FU 6–12 months	41 (100)	30 (73)	11 (27)	
GOS 6–12 months				
Moderate disability	6 (15)	2 (7)	4 (36)	0.035
Good recovery	35 (85)	28 (93)	7 (64)	
Number of patients at FU 12–18 months	50 (100)	39 (78)	11 (22)	
GOS 12–18 months				
Moderate disability	5 (10)	3 (8)	2 (18)	NS
Good recovery	45 (90)	36 (92)	9 (82)	

*FU* follow-up, *GOS* Glasgow Outcome Score, *NS* not significant

## Discussion

Despite the differences in ASA classification and preoperative KPS, there was no significant difference in mortality, complication rate and long-term outcome between younger and older (65 +) adults after surgery for meningioma. As expected, the postoperative clinical outcome 6–12 months after surgery was poorer for 65 + than for younger adults, but in patients with a follow-up between 12 and 18 months, this difference was no longer present.

ASA-classification is one of the most commonly identified factors associated with mortality after surgery for meningioma [13]. Compared to adults, Bateman et al. found that patients over 70 years old are three times as likely to die in the hospital, but conflicting data for 65 + have been reported [6–9, 12, 17, 19, 26–42]. In our cohort, the overall 30-day mortality was 2%, which is comparable to results of recent literature. We found no difference between age groups. Poon et al. found poorer functional outcomes in the older patients at 12 months’ follow-up compared to younger adult patients [28]. Our study suggests the same for 6–12 months’ follow-up, but 12–18 months after surgery clinical outcome was the same for both age groups. This emphasizes the value of longer-term follow-up, providing more insight into recovery time for younger and older adults.

In the literature, data comparing the destination after discharge in older meningioma patients compared to younger adults are limited. Bateman et al. described that older patients were five times more likely to have an adverse outcome, which was defined as either death or discharge to a facility other than home [6]. These findings were reported as independent effects of age, after correction for comorbid medical conditions. Unfortunately, it was not possible to compare our data with these results because ‘death’ and ‘discharge to other facility than home’ were combined in a single outcome parameter in Bateman’s study. It was not possible to reproduce the specific data on discharge destination. Poon et al. also found that adult patients were discharged to home more often than older patients [28]. Our results confirm this finding. Furthermore, we found that most of the older subjects could not go home straight after surgery, but could go home when minor home care was organized. The same is found after general surgery for older adults. Alexander et al. described that 50.8% of 592 patients ≥ 75 years old were discharged home with skilled services or to a skilled nursing facility after abdominal and pelvic cancer surgery [43]. Length of hospital stay after non-elective general surgery is found to be longer for older patients compared to younger adults [44, 45]. Simmonds et al. studied colorectal surgery in elderly patients. After studying 34,194 subjects, they found that elderly patients had an increased frequency of comorbid conditions and the incidence of postoperative morbidity and mortality increased progressively with advancing age. However, they concluded that selected elderly patients benefit from surgery since a large proportion survive for at least 2 years, irrespective of their age [46].

Taking into account the differences in course of recovery and destination after discharge, the results of our study suggest that meningioma surgery is well tolerated in patients at high age (both 65 + and 70 +). Thus, age alone need not be a contraindication for surgery. Clinical judgement and operative risk assessment (for which tumor location and degree of tumor vascularity is extremely important) remain to play an important role in the decision-making for surgery. After surgery, older patients need close follow-up and liberal use of rehabilitation, since they need more time to recover from meningioma surgery.

Some shortcomings of this study need to be addressed. All data were retrospectively collected from medical records, and unfortunately some follow-up data for 6–12 months and 12–18 months were missing or inconsistent. These data were missing because (part of) follow-up took place at the medical center that referred the patient in the first place. There is no reason to assume that the missing data are from patients who performed better or worse than the others. A comparison between the patients who were, and were not, assessed at 6–12 months and 12–18 months follow-up, revealed no significant differences in terms of gender, preoperative KPS, ASA classification, WHO grade and neurosurgical- and systemic complications after surgery. Since the missing follow-up data are missing at random, the results for 6–12 months and 12–18 months after surgery seem representative, although they must be interpreted with caution.

Another shortcoming of this study is the influence of comorbidity. Ideally it would be interesting to compare the two groups of patients after adjustment for comorbidity using for instance regression modeling, but unfortunately the sample size was too small for this adjustment. Therefore, we performed a subgroup analysis per ASA classification for the variables in Tables 2 and 3. This subgroup analysis showed a significant difference in discharge location for ASA II patients. For this ASA group duration of hospital stay and KPS at 12–18 months also turned out to be significantly different between the age groups. ASA II subjects ≥ 65 years old stayed in the hospital longer and had a lower KPS at 12–18 months compared to younger patients. These differences are not present in the overall analysis (Table 3). Since patient groups are very small for different ASA categories, it is hard to draw conclusion from this subgroup analysis.

As mentioned, a major limitation of this study is the small sample size, which may influence the reliability of the results. Larger series, or pooling of high-quality small series, are needed to confirm our findings.

## Conclusion

Postoperative outcome 30 days and 6–12 months after meningioma surgery, in terms of discharge destination and GOS score, was worse in older surviving patients compared to younger adults. At 12–18 months follow-up, however, older patients reached the same level of recovery as younger adult patients. Complication rate and mortality showed no differences between both age groups during the whole follow-up period.

As in all patients, careful patient selection is essential to reach good results in 65 + patients when considering meningioma surgery. Moreover, it is important to inform those patients and their relatives about the potentially longer period of recovery and temporary discharge to another care facility.

## Abbreviations

WHO: World Health Organization
KPS: Karnofsky Performance Status
ASA-classification: American Society of Anesthesiologists Physical Status Classification
GOS: Glasgow Outcome Scale
